# Dependency-dependent interference: NPI interference, agreement attraction, and global pragmatic inferences

**DOI:** 10.3389/fpsyg.2013.00708

**Published:** 2013-10-07

**Authors:** Ming Xiang, Julian Grove, Anastasia Giannakidou

**Affiliations:** Linguistics Department, University of ChicagoChicago, IL, USA

**Keywords:** memory interference, pragmatic inference, individual differences, autistic traits, NPI licensing

## Abstract

Previous psycholinguistics studies have shown that when forming a long distance dependency in online processing, the parser sometimes accepts a sentence even though the required grammatical constraints are only partially met. A mechanistic account of how such errors arise sheds light on both the underlying linguistic representations involved and the processing mechanisms that put such representations together. In the current study, we contrast the negative polarity items (NPI) interference effect, as shown by the acceptance of an ungrammatical sentence like “*The bills that democratic senators have voted for will **ever** become law*,” with the well-known phenomenon of agreement attraction (“*The key to the cabinets are …* ”). On the surface, these two types of errors look alike and thereby can be explained as being driven by the same source: similarity based memory interference. However, we argue that the linguistic representations involved in NPI licensing are substantially different from those of subject-verb agreement, and therefore the interference effects in each domain potentially arise from distinct sources. In particular, we show that NPI interference at least partially arises from pragmatic inferences. In a self-paced reading study with an acceptability judgment task, we showed NPI interference was modulated by participants' general pragmatic communicative skills, as quantified by the Autism-Spectrum Quotient (AQ, Baron-Cohen et al., 2001), especially in offline tasks. Participants with more autistic traits were actually less prone to the NPI interference effect than those with fewer autistic traits. This result contrasted with agreement attraction conditions, which were not influenced by individual pragmatic skill differences. We also show that different NPI licensors seem to have distinct interference profiles. We discuss two kinds of interference effects for NPI licensing: memory-retrieval based and pragmatically triggered.

## Introduction

During the processing of long distance dependencies, sometimes an element in a sentence that should be irrelevant for constructing a dependency interferes—a phenomenon that has been dubbed the “interference effect.” For instance, agreement attraction errors, such as ^*^the key to the cabinets are …, involve an agreement dependency between the singular subject *the key* and the plural copula verb *are* which is ungrammatical because of a number mismatch. However, the intervening noun “*cabinets*” interferes, facilitating the processing of the ungrammatical sentence^1^. Facilitation effects from an interfering element have been shown by various processing measures: such sentences are relatively common in spontaneous production; they can be elicited in controlled laboratory experiments; they are judged to be relatively acceptable; and online reading times on the otherwise problematic verb are generally reduced compared to number mismatched verbs without interference (Bock and Miller, 1991; Bock and Eberhard, 1993; Pearlmutter et al., 1999; Eberhard et al., 2005; Wagers et al., 2009; Dillon et al., 2013).

Such interference effects have been explained as instances of memory interference triggered during cue based memory retrieval. During incremental parsing of a long distance dependency, the tail of the dependency initiates the retrieval of the head in memory. This retrieval is prone to interference when the intermediately preceding material shares certain features with the set of retrieval cues that the parser employs (McElree et al., 2003; Lewis and Vasishth, 2005; Lewis et al., 2006; Wagers et al., 2009). Memory interference can be driven by partially matched morpho-syntactic features, as has been repeatedly shown by agreement attraction errors like the example above, and examples like “*The new executive who oversaw the middle managers were dishonest*” (example from Dillon et al., 2013). In such cases, memory retrieval is initiated in order to search for a plural subject at the plural verb, e.g., “were.” Because the search mechanism is content addressable (McElree et al., 2003), it may target any item in memory during the search process, leading to erroneous acceptance of interfering material which bears feature similarity to the correct retrieval target.

A large body of the research on interference effects has focused on deriving a thorough mechanistic account of the errors people make in interference situations, as such an account helps in constructing a precise parsing algorithm for long distance dependencies. But the pursuit of a domain-general parsing mechanism has somewhat overshadowed the question of whether or not different kinds of linguistic dependencies should be handled by the same parsing algorithm, and hence whether or not interference arises in the same way across different dependency types. One reasonable hypothesis is that the precise nature of a particular type of linguistic dependency is relevant to explaining differences in how dependencies are processed. In the current study, we tackle the question of whether or not there are dependency-dependent interference effects by looking at a case of interference that seems very much like agreement attraction on the surface, but that may at least partially arise from a different underlying mechanism. The particular type of interference we will discuss appears in the licensing of negative polarity items (NPIs), as in the sentence “^*^The documentaries that no network TV stations have played during prime time have ever been very controversial,” where the presence of *ever* is illicit. We argue that although such interference may superficially look the same as the subject-verb agreement errors discussed above, there are actually multiple different sources that contribute to NPI interference. In particular, in addition to a memory-retrieval based interference that is similar to agreement interference, there is also a separate rout of pragmatic inferences made at the message level during semantic integration. A sufficient account of NPI interference needs to take into account the close interaction between grammar (e.g., syntax and semantics) and pragmatic inference in sentence processing.

### NPI licensing and interference

NPIs are lexical items that need to be licensed in an environment that possesses a particular logical-semantic property. Negation is a cross-linguistically attested licensor for NPIs (as noticed in Klima, 1964). Licensing typically requires the NPI to be in the semantic and syntactic scope (i.e., c-command domain) of negation (Ladusaw, 1979, 1980; Giannakidou, 1998, 2011 for an overview). As shown in (1), the NPIs *any* and *ever* are grammatically licensed when they appear within the scope of negation (1a, b), but they are ungrammatical when there is no negation present (1c, d), or when negation is present, but doesn't c-command the NPI (1e, f).

(1) a. John *didn't* talk *to anybody*.
b. John *hasn't ever* talked to Bill.
^*^c. John has *ever* talked to Bill.
^*^d. John talked to *anybody*.
^*^e. *Anybody didn't* talk to John.
^*^f. The debate that *nobody* cared about will *ever* end.

Because of their apparent sensitivity to the presence of negation, *any* and *ever* are labeled “negative” polarity items (NPIs)^2^, but it must be noted that their distribution, and that of similar NPIs crosslinguistically, is quite broad and includes a vast range of negative and non-negative licensors, including conditionals, modal verbs, generic sentences, questions, the scope of universal quantifiers, comparatives, disjunctions (see Giannakidou, 2011 for detailed overview). Given this broad distribution and the potential differences among NPI classes in English and crosslinguistically, what semantic property unifies licensors as a natural class has been a matter of intense study—and researchers generally agree that NPIs appear in non-veridical environments. Non-veridical environments are (a) negative environments with negation and negative quantifiers (Baker, 1970a; Linebarger, 1980), (b) downward entailing environments (Ladusaw, 1980; Zwarts, 1986, 1996; Hoeksema, 1994; von Fintel, 1999, inter alia), and (c) other non-veridical environments that may not be negative or downward entailing (e.g., modal expressions, questions, imperatives, generic statements; Zwarts, 1995; Giannakidou, 1998, 2006, 2011; Bernardi, 2002). We cannot provide a detailed survey here; but as background for the specific data we address, we discuss licensing by negation and downward entailment (DE) in the next section^3^.

In the domain of NPI licensing, an “interference effect” is said to result when an unlicensed NPI becomes more acceptable if a licensor is inserted into the preceding context—but crucially, is *not* in the right structural (c-commanding) position (Drenhaus et al., 2005; Vasishth et al., 2005, 2008). In the example below (examples taken from Drenhaus et al., 2005), the expected contrast obtains between (2a) and (2b); however, there is also a significant difference between the ungrammatical sentences (2b) and (2c). (2c) is judged as “more acceptable” than (2b), even though the licensor *no* doesn't c-command the NPI *ever*, so that it should be unlicensed.

(2) a. **No** man who had a beard was *ever* happy.
^*^b. A man who had a beard was *ever* happy.
^*^c. A man who had **no** beard was *ever* happy.

In the (c) example, negation is present but not in a position c-commanding *ever,* as is required for licensing. The NPI *ever* therefore remains unlicensed. In online measures, NPI interference effects have appeared as facilitatory effects (e.g., shorter RTs or smaller ERP amplitudes) on the problematic NPI in the interference condition, as compared to the NPI in the condition with no licensors anywhere in the sentence (Drenhaus et al., 2005; Vasishth et al., 2008; Xiang et al., 2009; Parker et al., 2013).

The interference effect above is on the surface very similar to the memory interference phenomenon introduced earlier for subject-verb agreement. One account of NPI interference is indeed couched upon retrieval interference due to feature similarity between the retrieval cues and the previously processed linguistic information. Vasishth et al. (2008) argued that the parser uses lexical semantic cues such as [+negative] and syntactic cues such as [+c-command] to retrieve a proper licensor for *ever* from previously processed material in memory. For (2b), no such match is found, and the sentence is determined to be unacceptable. For (2c), however, the quantifier *no* in the embedded subject position partially matches the search criteria: although it doesn't match the syntactic cue [+c-command], it does satisfy the cue [+negative]. During retrieval of a licensor, this partial feature match may boost the activation level of the memory representation of the embedded quantifier *no*, causing it to be more likely to be retrieved once its activation level goes beyond a certain threshold.

Although such an account is plausible, as well as parsimonious, we think it falls short of providing a complete account of NPI interference, because it misses some important distinctions between NPI licensing, on the one hand, and syntactic dependencies such as subject-verb agreement and those involved in relative clauses and cleft constructions, on the other hand. Specifically, while the latter dependencies types involve syntactic relations between lexical items (e.g., a subject and an agreeing verb, or a head noun and a verb in a relative clause), NPI licensing involves not only syntactic conditions (e.g., the c-command requirement on a proper licensor, but also see our remarks in the general discussion about this syntactic condition), but also logical-semantic (e.g., negation, DE, non-veridicality), and pragmatic conditions. Crucially different from subject-verb agreement, pragmatic inferences derived from global semantic interpretation (which traditionally have been considered outside of the grammar proper), can be used to license NPIs (Linebarger, 1980, 1987; Giannakidou, 1998, 2006). We will discuss the pragmatic licensing mechanism in more detail in the next section.

Closely related to the fact that NPI licensing involves multiple mechanisms, there are many different types of licensors other than just the negative determiner *no*, which has been the focus of most of the studies on NPI interference. Interference under the licensor *no* may look superficially similar to subject-verb agreement, because one can identify a [+negative] feature on the licensor, which, when served as a memory retrieval cue, may lead to interference. Whether or not this is indeed the underlying mechanism, or only one of the mechanisms involved, is an empirical question we will address in this paper, but a cue-driven process is at least a logical possibility here. Importantly, when we look at a larger set of licensors, postulating a lexical [+negative] feature becomes untenable for many of them: for instance, with a universal quantifier *every*, focus *only*, conditional *if*, emotive factives like *surprised, amazed*, etc. We focus on *only* here, since it can be used as a determiner and therefore constitutes a minimal pair with *no*. We assume that *only* licenses NPIs through a negative exceptive component, since a sentence of the form “[Only NP] VP” entails “[Nobody other than NP] VP” (see the discussion in the section below). But there is little reason to believe that *only* itself contains in its lexical entry a grammatical/syntactic [+negative] feature. Klima (1964) gave syntactic diagnostics for syntactically negative expressions, which include phonologically and morphologically negative expression such as *no, none, never*, but also negative expressions that are not overtly marked in morphology or phonology as negative, such as *few, scarcely, hardly, seldom, rarely*, etc. For example, all of these expressions can be followed by a conjunct with a *neither*-tag, but not by *so*-tag; they may also co-occur in a conjunct with *either*, but not with *too*; etc. We provide some examples below, showing that *only* is not a negative expression under these syntactic diagnostics. Nor are the other non-negative licensors we mentioned above, as the reader may verify.

(3) a. Publishers will usually reject suggestions, and no/few/^*^only/^*^a few writers will accept them, ***either***.
b. Publishers will not/hardly/seldom/rarely/^*^only/^*^usually accept suggestions, and ***neither*** will the writers.

In the absence of a lexically coded [+negative] feature that can trigger a similarity-based interference effect (as with *only*), the question arises whether or not we will still see interference, and, if we do see such an effect, what would account for it. We will address these questions in the current study by examining both the licensors *only* and *no*.

### Flexibility of licensing with english NPIs: the role of pragmatic inferencing

It has long been observed that some NPIs become licensed even when no grammatical lexical licensors are present on the surface that contain the required logical-semantic property for licensing. The following examples are largely taken from Linebarger (1987):

(4) a. John kept writing novels *long after* he had *any* reason to believe they would sell.
b. *Exactly four* people in the world would have *ever* read that dissertation: Bill, Mary, Tom, and Ed.
c. Mary was *surprised* there was *any* food left.
d. I am *sorry* that I ever met him.
e. *Only* the students who have *ever* read anything about phrenology attended the lectures. [=117 in Ladusaw (1980)]

In all these examples, there aren't any explicit lexical items that can serve as grammatical licensors, in the sense that they possess the logical property necessary for licensing. Surely, the items *long after, exactly four, amazed*/*surprised*, and *only* are responsible for the appearance of *any* and *ever*, but they are not logically negative, nor DE, nor non-veridical. Consider the property of negation/DE. DE expressions, as is traditionally stated, allow logical inferences in their scope from a set to a subset. Consider the following entailment relations with negation [examples adapted from Linebarger (1987)].

(5) a. John didn't eat a green vegetable for dinner.
b. John didn't eat kale for dinner.

Kale is a subset of green vegetables. If John didn't eat a green vegetable for dinner (5a), it logically follows that John didn't eat kale for dinner (5b). The superset-to-subset logical inference is the hallmark of negative and DE expressions. Ladusaw proposed that NPIs appear in the scope of negative and other DE expressions (such as negative quantifier *few*, or the restrictor of universal quantifier *every*). However, none of the examples in (4) contains a DE expression. We show below on this point for “*only*” and “*long after*” [see Linebarger (1987); Atlas (1993); Horn (1996); von Fintel (1999) and Giannakidou (2006) for more discussion that *only* and emotive factive verbs such as *sorry* and *surprise* are not DE in its strict sense].

(6) a. Only Bill went to Greece;
b. Only Bill went to Athens.
(7) a. Bill married Samantha long after he travelled to Europe.
b. Bill married Samantha long after he travelled to France.

We see here that the subset inference from (a) to (b) sentences in (6) and (7) is not licensed with the critical expressions “only” and “long after,” and they are therefore *not* logically DE^4^.

Faced with many examples of this kind, in which a grammatical licensing mechanism relying on the logical properties of a licensor does not seem to suffice, several researchers have advanced proposals to distinguish a pragmatic licensing mechanism from a grammatical (syntactic/semantic) one. Giannakidou (1998, 2006), for example, talks about two modes of licensing, one semantic, relying on a (c-commanding) grammatical licenser (“direct” licensing), and another, “global pragmatic” licensing (“indirect” licensing) that relies on the availability of a negative inference. In the regular case, NPIs are licensed directly by an expression that bears the required logical-semantic property. However, in the absence of such a grammatical licensor, either the use of an NPI leads to ungrammaticality, or the context enables comprehenders to derive a negative inference pragmatically, which in turn licenses NPIs (see also Baker, 1970a,b; Linebarger, 1987). Such pragmatic inferences have been called “implicatures,” and we will refer to them as such from now on^5^. Linebarger (1987) and Giannakidou (2006) have considered *only* as a candidate for pragmatic licensing. The basic intuition there is that the exclusive component in the meaning of *only* is responsible for licensing NPIs (e.g., *Only John ate kale* entails that *Nobody other than John ate kale*). In our recent work (Xiang et al., 2012, 2013), based on ERP evidence, we argued that *only* is a semantic licensor that licenses NPIs through negation in the asserted content (see Atlas, 1993; Horn, 1996, for the semantics of *only*). Although they are different in their specific details, none of these proposals treat *only* as a negative expression that contains a lexically coded [+negative] feature, keeping in line with our discussion in the last section.

It is also crucial to note that, although licensing through global pragmatic reasoning is a possible mode of licensing for many NPIs, not all negative implicatures can be used to make NPIs acceptable (Linebarger, 1987; Horn, 1989, 2002; Giannakidou, 2006). For instance, “*almost”*—though clearly inviting a negative inference (*John almost finished the book* implies that *he did not finish it*)—does *not* license NPIs: ^*^John almost finished anything^6^. Although the boundary between inferences that can and cannot license NPIs is still an open question, we follow the suggestion in Linebarger (1987) and assume that in order for a derived negative inference to be able to license NPIs, it should be prominent in the sense that the derived proposition warrants the truth of the original proposition. Consider our earlier example with *long after*:

(8) a. John kept writing novels *long after* he had *any* reason to believe they would sell.
b. John kept writing novels even though he didn't have any reason to believe they would sell.

The NPI *any* in (8a) is licensed under the derived negative implicature (8b). There is a very strong inference to (8b) from (8a), and in fact the two are almost semantically equivalent. Most important, if (8b) is true, then it is also true that *John kept writing novels long after he had any reason to believe they would sell*. It seems, then that a “useful” negative inference is one that is semantically close enough to the original proposition. How to formally quantify the notion of “semantic closeness” is an open question and is beyond the scope of the current discussion. What is crucial for current purposes is, first that negative implicatures provide a possible licensing mechanism, at least in English; and second, not all negative implicatures can license NPIs. It is possible that the difference between the “useful” and “useless” inferences is a categorical one, but it is also possible that the two simply occupy different ends of a continuum of pragmatic inferences, on which one finds different degrees of “licensing strength.” We will leave this question open. We turn below to the empirical focus of the current paper: the case of NPI interference. We will argue that the interference observed in NPI licensing is at least partially driven by the over-application of the pragmatic licensing mechanism. That is, in cases of NPI interference, comprehenders resort to the pragmatic strategy, i.e., they attempt to use a pragmatic inference, which, however, cannot properly license NPIs. The effect is that such an illicit interference will occasionally boost the acceptability of unlicensed NPIs. The availability of such pragmatic inferences, as we will show, is modulated by individual subjects' pragmatic skills.

### Interference driven by pragmatic inference

Xiang et al. (2009) argued that the NPI interference effect stems from over-application of a flexible, inference-based licensing mechanism that is already in place in the grammar. One possibility, as suggested in Xiang et al. (2009), is that, while parsing a statement like “*the bills that no democratic senators have voted for will* P” (“P” stands for an upcoming predicate), people generate a negative inference about a contrasting set of referents “*the bills that democratic senators HAVE voted for will NOT have the same property P*” on some proportion of trials. Note that such an inference is not logically valid, nor can it be derived from any proper grammatical device. But the particular construction involved in NPI interference effect, i.e., relative clauses, may be responsible for triggering such negative inferences. It is known that restrictive modifiers generally invite inferences about a contrastive set of referents pragmatically (Altmann and Steedman, 1988; Tanenhaus et al., 1995; Sedivy et al., 1999). It has been shown that people are very sensitive to the pragmatic cues of restrictive modifiers: restrictive modifiers perform a discourse function to distinguish the set of referents that possess the property described by the modifier and the set that do not. Such discourse principles are active in parsing because interlocutors engaged in a discourse interaction adhere to the general communicative principle that the exchange of information should be as informative as it needs to be (Grice, maxim of quantity, 1975). To our knowledge, almost all studies on NPI interference so far in the literature have used restrictive relative clauses to host an “intruding” licensor. It is plausible then to argue that the choice of this particular structure facilitates the triggering of negative inferences about a contrasting set.

Although pragmatic inferences driven by communicative pressure are very common in natural language communication, they in general are not qualified to actually license NPIs. If we adopt our rudimentary notion of “semantic closeness” in the last section, the negative inferences made in the interference scenarios are not “close” enough to the original propositions. Consider again the interference example “*The bills that no democratic senators have voted for will become law*.” The potential negative inference “*The bills that democratic senators have voted for will NOT become law*” does not have similar enough truth-conditions to the original proposition. Not being semantically close, the negative inference is too weak to render NPIs totally acceptable. But since pragmatic inferences may in principle license NPIs in English, comprehenders may over-apply this mechanism and use it in some proportion of the ungrammatical trials, so that negative inferences that are normally not useful for NPI licensing have a facilitating effect on acceptability.

If the interference effect with NPIs is due to over-application of pragmatic inferences, in which subjects extract a negative implicature from the given context, we predict that NPI interference effects should be modulated by individual participants' ability to extract pragmatic inferences from context. Different individuals may possess varying abilities to carry out complex pragmatic reasoning, and we hypothesize that participants who are better at pragmatic reasoning will be more prone to an NPI interference effects, since it is more likely for these participants to successfully construct negative inferences from context, making them more vulnerable to over-applying the pragmatic licensing mechanism. On the other hand, participants who are less skilled in pragmatic inference will generate fewer inferences, and these participants will be more likely to avoid the interference effect.

Furthermore, in the current study, we compare NPI interference with a purely syntactic dependency: subject-verb agreement. We predict that, if the correlation between pragmatic skills and interference in NPI licensing is driven by over-application of a pragmatic-licensing mechanism that is specific to NPIs, no similar correlation should hold between the magnitude of the agreement interference (attraction) effect and individual pragmatic differences, despite the superficial similarity between NPI interference and agreement attraction errors. We test these predictions in the current study. Individual pragmatic skills of our participants were assessed and quantified by the autism-spectrum quotient (AQ, Baron-Cohen et al., 2001), which we turn to now.

### The autism-spectrum quotient

Pragmatic language problems are among some of the defining characteristics of children and adults with autism (Bishop, 1989; Tager-Flusberg et al., 2005). For example, their linguistic behavior may often consist in inappropriate comments; and they may have difficulty comprehending jokes, sarcasm, and indirect requests (Happé, 1993; Ozonoff and Miller, 1996; Wang et al., 2006). However, it is increasingly recognized that autistic traits are likely to be present on a continuum among the general population, and people who are diagnosed as autistic simply represent one end of this continuum. This raises the possibility that even among the neurotypical population, there exist individual pragmatic differences associated with individual autistic traits. The AQ (Baron-Cohen et al., 2001) assesses the extent of autistic traits that neurotypical individuals possess. There are a total of 50 questions, divided into 5 subscales, each with 10 statements, to which the subject must reply with one of the choices: “Definitely agree,” “Slightly agree,” “Slightly disagree,” or “Definitely disagree.” The 5 subsets of questions are designed to tap into five different cognitive functions that have been found to be important when characterizing autistic behavior. The five subscales and a corresponding example item are: social skills (e.g., “I prefer to do things with others rather than on my own.”); communication (e.g., “Other people frequently tell me that what I've said is impolite, even though I think it is polite.”); attention to detail (e.g., “I often notice small sounds when others do not.”); imagination (e.g., “If I try to imagine something, I find it very easy to create a picture in my mind.”); attention switching (e.g., “I prefer to do things the same way over and over again”). Half of the questions are designed to elicit an answer of “definitely agree” or “slightly agree”; and the other half, “definitely disagree” or “slightly disagree.” Baron-Cohen et al. (2001) provide scoring guidelines. Higher scores indicate more association with autistic traits.

There is an increasing number of studies that document the correlation between AQ (or AQ subscale) scores and processing in certain specific linguistic domains among the neurotypical population (Stewart and Ota, 2008; Nieuwland et al., 2010; Yu, 2010). Particularly relevant for current purposes, the communication and social skills subscales have been linked to pragmatic language comprehension; in particular, the processing of scalar implicatures (Nieuwland et al., 2010; Sikos et al., 2013) and perspective taking (Grodner et al., 2012). For example, Nieuwland et al. (2010) showed that when computing scalar implicatures (e.g., *some* implies *not all*), participants' ability to generate scalar implicatures online was significantly correlated with their communication subscale scores (CS scores). In particular, participants with better communication skills (i.e., lower CS scores) were more likely to access the scalar implicature interpretation of a sentence like “*some elephants have trunks*,” and consequently detect the anomaly of under-informativity.

The growing body of work that shows a correlation between AQ scores and pragmatic language skills makes the AQ a suitable tool for the current study to probe the underlying differences between NPI and subject-verb agreement dependencies. Admittedly, such a correlation only provides a classificatory diagnostic, rather than an explanation of the mechanisms underlying pragmatic reasoning, since it is not yet clear how the communicative and social skills measured in the AQ are recruited in language comprehension. Although the exact nature of the link between extra-linguistic skills and linguistic pragmatic reasoning is not well-understood, the link itself is nevertheless supported by empirical evidence, suggesting that the same cognitive mechanisms may be shared between the two types of tasks. Thus, the AQ provides us with a way to operationalize individual differences in pragmatic reasoning.

## Current experiment

### Method

#### Materials

There are two types of target items in this study: NPI and subject-verb agreement. Table 1 gives an example of each type. For the NPI materials, there are three basic types of conditions. In the ***Licensed*** conditions (9a and 9b), the NPI *ever* is licensed by a grammatical licensor. In the ***Interference*** conditions (9c and 9d), *ever* is not licensed properly: even though there are licensors (*no* and *only* again) in the same context, they are not in a syntactically c-commanding position. Finally, the ***Plain Unlicensed*** conditions contain unlicensed NPIs with no potential licensors in the preceding context.

**Table 1:** **Example stimuli**.

**(9) NPI**	
a/c Licensed	***No***/*only* documentaries that the network TV stations have played during prime time have ***ever*** been very controversial.
b/d Interference	The documentaries that ***no***/***only*** network TV stations have played during prime time have ***ever*** been very controversial.
e Plain Unlicensed	The documentaries that the network TV stations have played during prime time have ***ever*** been very popular.
**(10) AGREEMENT**	
a Grammatical	The ***receptionist*** who the ***boss*** depends on never ***fails*** to do a stellar job.
b Interference	The ***receptionist*** who the ***bosses*** depend on never ***fail*** to do a stellar job.
c Plain Ungrammatical	The ***receptionist*** who the ***boss*** depends on never ***fail*** to do a stellar job.

For the Licensed and the Interference conditions, we looked at the two licensors *no* and *only* in this study to test the generality of previously observed interference effects. *Only* is different from *no* in at least two ways: first, as discussed earlier, *only* does not contain a [+negative] feature; second, *only* is much less frequent than *no* as a licensor (Xiang et al., 2009). This raises the question whether or not interference will arise for *only*, and, if so, whether or not the same account should apply to both licensors.

The set of agreement items (10a–c) was created using the same design. In the ***Grammatical*** condition, the main verb agrees with the matrix subject (*the receptionist*) in its singular number. In addition, the embedded subject (*the boss*) is also singular, creating no interference. In the ***Interference*** condition, the matrix verb fails to agree with the matrix subject, since the matrix subject is singular whereas the verb is plural. However, the intervening embedded subject also carries plural number, and hence may be incorrectly accepted as being in an agreement relation with the main verb. Finally in the ***Plain Ungrammatical*** condition, the main verb fails to agree correctly with the singular matrix subject, but the embedded subject is also singular, mismatching the main verb.

There were 60 sets of the NPI items, 40 sets of the agreement items, as well as 38 extra fillers. The items were distributed into multiple lists using a Latin square design, such that no participant was presented with more than one condition from the same item set.

#### Participants and procedure

Ninty-two native English speakers (mean age = 20, *sd* = 3.2, 52 female, 40 male) from the University of Chicago campus and surrounding area participated in the study for $10 payment or course credit. Each participant finished a self-paced reading task and also completed an AQ questionnaire (see below). The self-paced reading task was presented using the Linger software (Doug Rohde, MIT). Participants read through each sentence word by word at their own pace. After the last word of each sentence, a question appeared that said: “Is this acceptable?” After participants pressed one of the two answer keys (Y or N) on the keyboard, they went on to the next trial. Practice trials were provided before the experimental session to familiarize participants with the task. Each subject also completed an AQ questionnaire either before or after they completed the self-paced reading task (in a random order).

### Data analysis and results

Among the 92 participants, one did not finish the AQ questionnaire, and his data was not included in any of the analyses below. Three additional participants were excluded from the analysis due to very low overall accuracy across the whole experimental session (<50% correct). For the rest of the 88 participants, we analyzed their acceptance rate results and their online reading times at the critical word *ever*. The grand average results are presented in Table 2.

**Table 2:** **Average acceptance rate and RTs on the critical word, presented separately for the NPI and the agreement items (with *sd* in the parenthesis)**.

	**NPI Licensing**				
	***No*-Licensed**	***No*-interference**	***Only*-Licensed**	***Only*-interference**	**Plain unlicensed**
Acceptance rate	0.87 (0.16)	0.25 (0.30)	0.81 (0.17)	0.27 (0.32)	0.16 (0.27)
RT at the CW (ms)	421 (114)	439 (120)	420 (120)	453 (146)	473 (155)
	**Agreement**				
	**Grammatical**	**Interference**	**Plain ungrammatical**		
Acceptance rate	0.92 (0.18)	0.28 (0.23)	0.12 (0.17)		
RT at the CW (ms)	463 (125)	509 (169)	547 (202)		

For the data analysis, we will present results from mixed effects logistic regression models on the acceptance rate data and results from mixed effects linear regression models on the reading times (Baayen et al., 2008). The models were constructed using the *lmer* function in the lme4 package in R (Bates et al., 2012). Separate analyses were carried out for each subset of the target materials (i.e., NPIs and agreement materials). All models reported here are maximal models that have converged (Barr et al., 2013). For the mixed effects models, main interest of comparisons were set up as contrasts with Helmert coding (Venables and Ripley, 1999; Vasishth and Broe, 2011; Vasishth and Drenhaus, 2011), and they were included in the mixed effects models as fixed effect predictors (see below). Since the CS from the AQ questionnaire was the major subscale that has been shown to reflect speakers' pragmatic reasoning abilities in language processing (Nieuwland et al., 2010; Sikos et al., 2013), we will mainly focus on this subscale of the AQ^7^. Each participant's CS score was entered into the mixed effects models as an additional fixed effect predictor. Random effect structure included random intercepts for subjects and items, as well as random slopes of the fixed predictors. Before constructing the models, reading times longer than 2000 ms were removed, and all reading times were log-transformed.

#### NPI licensing

***Acceptance rate.*** The averaged acceptance rate of each condition is presented in Table 2. As expected, the two licensed grammatical conditions (9a and 9c) have the highest acceptance rate (0.87 and 0.81), the unlicensed ungrammatical condition (9e) has the lowest acceptance rate (0.16). Critically, the interference conditions (9b and 9d) were accepted more often than the non-interference ungrammatical one (0.25 and 0.27), manifesting a standard interference effect.

We first analyzed all the data together, using a mixed effects logistic model. We defined three orthogonal contrasts: the first contrast examined the grammaticality effect (***Grammaticality***), in which the licensed grammatical conditions were contrasted with the ungrammatical conditions (i.e., *a, c* vs. *b, d, e*,); the second contrast examined the interference effect (***Interference***), in which the interference ungrammatical conditions *b* and *d* were contrasted with the unlicensed ungrammatical condition *e* (*b, d* vs. *e*); in the third contrast (***Licensor***) the two types of licensors were compared (*a, b* vs. *c, d*). These three contrasts were entered into the mixed effects model as fixed effect predictors. In addition, we included each participant's CS scores from the Autism Quotient as another fixed effect predictor in the model. Among the 88 participants included in this analysis, the minimum CS score was 0 and the maximum was 10, with a mean of 3.1 (median 3, and standard derivation 2.2). For the random effect structure, we included random intercepts for both subjects and items, as well as random slopes of the three user-defined contrasts above. The model output is presented in Table 3 below:

**Table 3:** **NPI licensing acceptance rate: fixed effects from the mixed effect logistic model for the overall data**.

	**Estimate**	**Std. Error**	***z* value**	***Pr*(>|*z*|)**
Grammaticality	5.08	0.66	7.74	9.63E-15^***^
CSscore	−0.11	0.08	−1.36	0.18
Interference	1.30	0.22	5.79	6.97E-09^***^
Licensor	0.65	0.30	2.20	0.03^*^
Gram:CSscore	0.08	0.17	0.47	0.64
Inter:CSscore	−0.13	0.06	−2.15	0.03^*^
Licensor:CSscore	−0.06	0.08	−0.81	0.42
Inter:Licensor	−1.03	0.77	−1.34	0.18
CSscore:Inter:Licensor	0.06	0.21	0.27	0.79

lmer[acceptance ~ gram * CSscore + interfence * CSscore + licensor * CSscore + licensor:interference + licensor:interference:CSscore + (1 + gram + interference + licensor|subj) + (1 + gram + interference + licensor|item), data = dataframe, family = “binomial ^8^”].

* p < 0.05;

*** p < 0.001.

Not surprisingly, the model revealed a significant effect for both Grammaticality and Interference. What is crucial, is that there is a significant interaction between the effect of Interference and CS scores, indicating that the difference between the interference condition on the one hand, and the plain unlicensed condition on the other, is affected by participants' general pragmatic skills assessed via their CS scores. In addition, there is also an effect of Licensor, suggesting a difference between the *no* and *only* conditions.

#### The effect of licensor type

**Figure 1:**
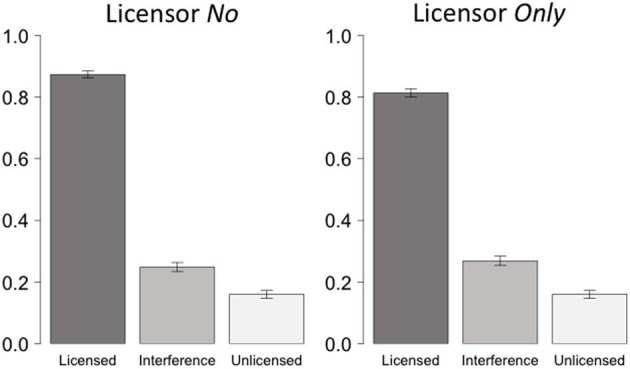
**Acceptance rate for the two different licensors**.

The data from licensor *no* and *only* are plotted separately in Figure 1. The unlicensed condition is shared by the two licensor groups (i.e., condition *e* in Table 1).

Paired comparisons between conditions showed that sentences licensed under *no* were accepted more often than those licensed under *only* (condition *a* vs. *c* in Table 1, *p* < 0.001); but the interference condition under *no* is not different from the interference condition under *only* (condition *b* vs. *d, p* > 0.2). Therefore, the effect of Licensor observed in Table 3 was mainly driven by the grammatical conditions: subjects were slightly more resistant in accepting *only* as a grammatical licensor. We will come back to this observation in the general discussion.

#### The interference-by-CS-scores interaction

Our model showed a robust interaction between the Interference effect and CS scores. We further discuss what this interaction entails in this section. Since our model revealed no interaction between CS scores and licensor type (i.e., neither three-way nor two-way interactions), we do not expect the effect of CS scores on interference to be conditioned by licensor type. For the completeness of our presentation, however, we present results from *no* and *only* separately.

In the analysis of licensor *no*, we only present the three relevant conditions in Table 1: conditions 9*a, b*, and *e*. The mixed effects model was constructed largely in the same way as before, except that only two contrasts were defined as fixed effect predictors: ***Grammaticality***, which contrasted the grammatical condition 9*a* with the other two ungrammatical conditions (i.e., *a* vs. *b* and *e*); and ***Interference***, which compared the interference ungrammatical condition 9*b* with the unlicensed ungrammatical condition *e* (*b* vs. *e*). For *only*, the three relevant conditions were 9*c, d*, and *e* in Table 1, and the two contrasts were defined as ***Grammaticality*** (*c* vs. *d, e*) and ***Interference*** (*d* vs. *e*). The model results for the fixed effects are presented in Table 4.

**Table 4:** **NPI licensing acceptance rate: fixed effects for two different NPI licensors**.

	**Licensor *No***	**Licensor *Only***
	**Pr(>|z|)**	***Pr*(>|*z*|)**
Grammaticality	4.2e-11^***^	6.9e-09^***^
Interference	1.6e-08^***^	6.2-07^***^
CSscore	0.15	0.37
Gram:CSscore	0.32	0.27
Inter:CSscore	0.03^*^	0.07^∧^

model = lmer[acceptance ~ gram * CSscore + inter * CSscore + (1 + gram + inter|subj) + (1 + gram + inter + CSscore|item), data = dataframe, family = “binomial”].

* p < 0.05;

*** p < 0.001;

∧ p < 0.1.

**Figure 2:**
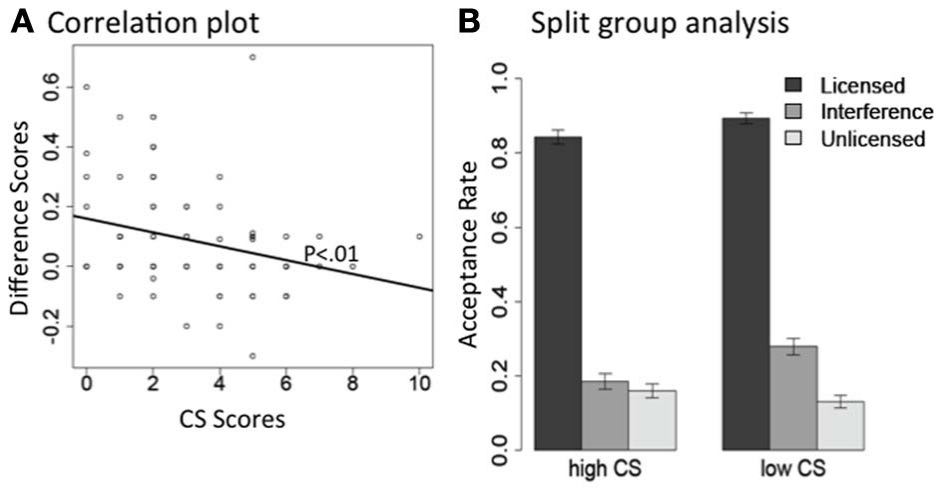
**The interaction between interference effect and CS scores in acceptance rate, for the NPI licensor *no*. (A)** Correlation between each individual subject's difference scores between their interference condition and the plain unlicensed condition (*Y*-axis: acceptance rate of Interference—acceptance rate of Unlicensed) and their CS scores (*X*-axis: CS scores). **(B)** Acceptance rate for each condition plotted separately for the high and low CS groups.

**Figure 3:**
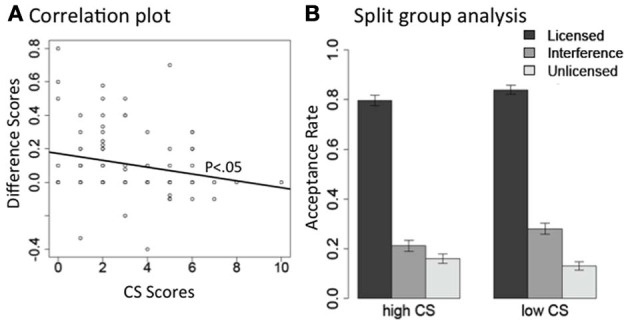
**The interaction between interference effect and CS scores in acceptance rate, for the NPI licensor *only*. (A)** Correlation between each individual subject's difference scores between their interference condition and the plain unlicensed condition (*Y*-axis: acceptance rate of Interference—acceptance rate of Unlicensed) and their CS scores (*X*-axis: CS scores). **(B)** Acceptance rate for each condition plotted separately for the high and low CS groups.

As expected, both the Grammaticality effect and the Interference effect are highly significant, and the interaction between Interference and CS scores is also significant. To better understand the interaction between CS scores and the interference effect, we did the following two analyses for licensor *no* and *only* separately. For each subset of the data, we first carried out a correlation analysis between the size of the interference effect and individual participants' CS scores. For each participant, we calculated a difference score between their acceptance rates, averaged across items, in the interference condition and the plain unlicensed condition. This difference score represents the size of the interference effect for each subject. We then correlated these difference scores with their CS scores. There is a significant *negative* correlation between the difference scores and participants' CS scores for licensor *no* [Pearson's *r* = −0.28, *t*_(86)_ = −2.7, *p* < 0.01], as well as for licensor *only* (Pearson's *r* = −0.21, *p* < 0.05). The negative correlation suggests that the higher a participant's CS score, the smaller the difference between their interference condition and plain unlicensed condition. In other words, participants with higher CS scores treated the interference conditions like the plain unlicensed condition, and rejected them both; on the other hand, participants with lower CS scores were more likely to erroneously accept the interference conditions. We plot the correlation results in Figures 2A, 3A.

Second, we carried out a split-group analysis. We separated our participants into two groups along the median split of their CS scores: participants in one group had CS scores above 3 (high CS group, *n* = 36), and participants in the other group had scores below 3 (low CS group, *n* = 43). Participants who had a CS of exactly 3 were not included in either group. In Figures 2B, 3B, we present the mean acceptance rate results for these two participant groups, separated by licensor type.

We carried out mixed effects models for each CS group under each licensor. Licensor *no* and *only* showed very similar patterns. For licensor *no* (Figure 2B), both high and low-CS groups showed the expected Grammaticality effect (*p*s < 0.0001); but only the low CS group showed an Interference effect (high CS: *p* > 0.3; low CS: *p* < 0.0001). For licensor *only* (Figure 3B), both high and low CS groups showed a clear Grammaticality effect (*p*s < 0.0001). The low CS group also showed a strong Interference effect (*p* < 0.0001), whereas this effect was much weaker for the high CS group (*p* < 0.06).

To summarize the acceptance rating data on NPIs, the group averaged data showed an interference effect, but this effect is crucially modulated by individual subjects' pragmatic-communicative skills, across different licensors.

#### Self-paced reading time

**Figure 4:**
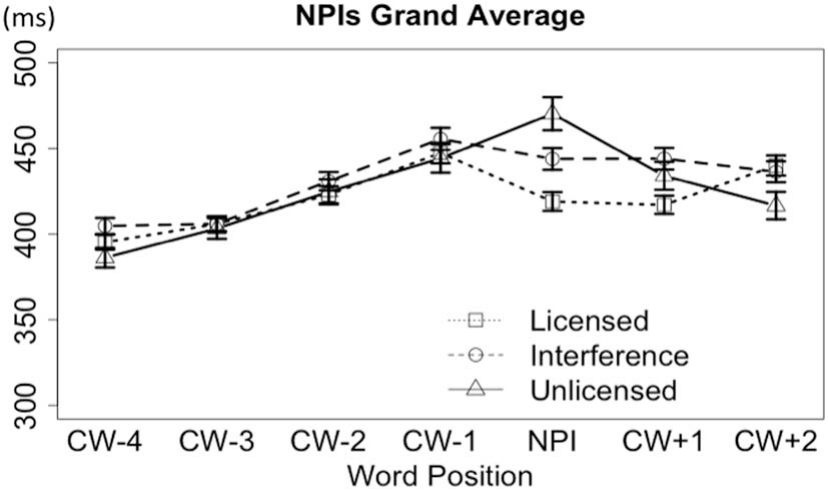
**Word-by-word reading times for the NPI stimuli set, collapsing the licensors “*no*” and “*only***.”

In Figure 4, we plot the reading time from four words prior to the NPI word *ever* and two words after it, with combined data from licensor *no* and licensor *only*. As shown in the plot, combined data from *no* and *only* showed differences among the licensed, interference, and unlicensed conditions only immediately at the critical NPI word (CW) *ever*. The grand average RTs on the CW are shown in Table 2.

We carried out mixed effects linear regression modeling on the RTs at the CW. The fixed and random effect structures are essentially the same as in our mixed effects logistic models discussed earlier. Prior to the analyses, we log-transformed all the RTs, and centered the CS scores. We first did analyses on the entire data set, and then did separate analyses for licensors *no* and *only*. The model output from the entire data set on the CW is presented below.

On the CW, the results revealed the expected effects for Grammaticality and Interference, but in contrast to the acceptance rate results, there was no interaction between CS scores and Interference. On the spill-over word CW + 1, there was a Grammaticality effect, but no Interference. There was also an unpredicted effect of Licensor. Further examination showed that this effect appeared because the grammatical and interference conditions under “only” were both read slower than the same two conditions under “no.” Since this effect wasn't predicted under any of our hypotheses, we will not go into it further. We next analyzed data for *no* and *only* separately.

#### Licensor no

**Figure 5:**
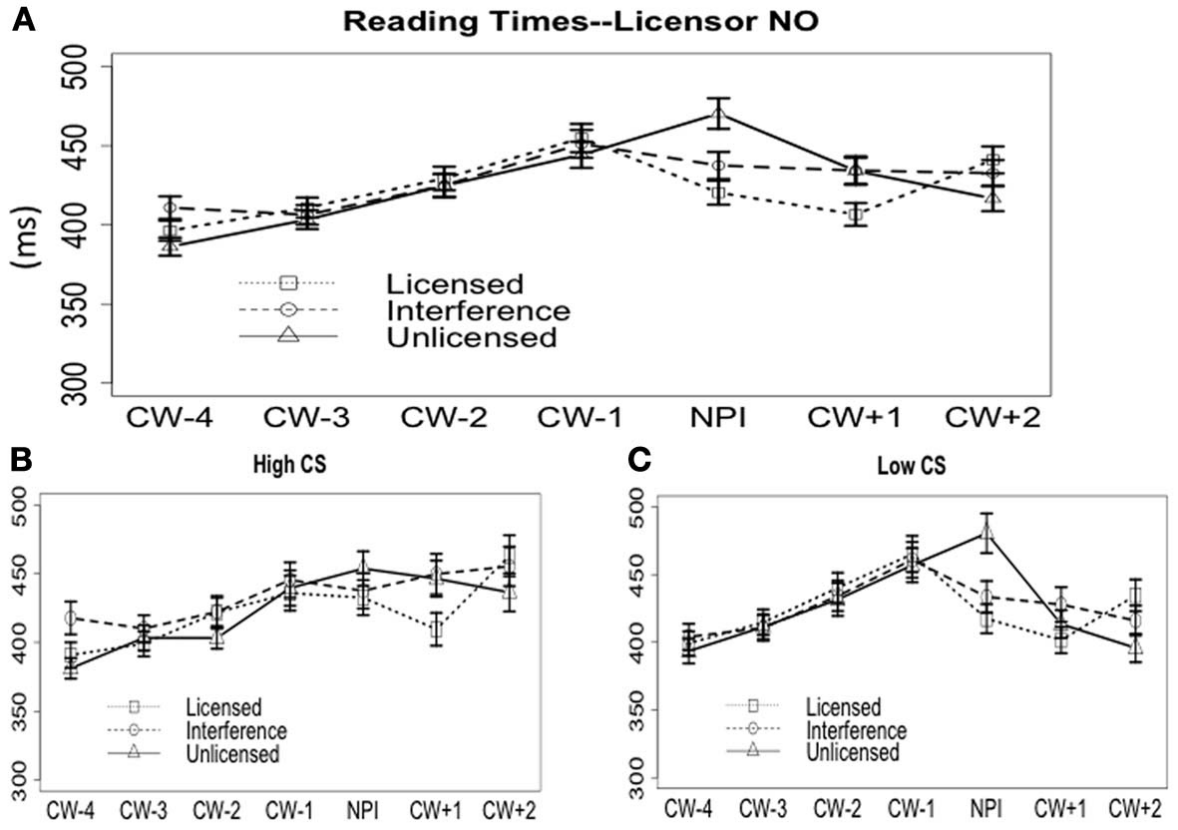
**Reading times when the NPI licensor was “*no.*” (A)** Word-by-word reading time cross all subjects. **(B)** Word-by-word reading time for the high CS group. **(C)** Word-by-word reading time for the low CS group.

***On the word CW.*** The word-by-word RTs (4 words prior and 2 words after the CW) are plotted in Figure 5A. On the CW *ever*, the grammatical condition was read faster (421 ms) than both the plain unlicensed condition (473 ms) and the interference condition (439 ms); but, the interference condition was also faster than the plain unlicensed condition, suggesting an interference effect.

The output of the mixed effects linear regression model for reading times on the CW is presented in Table 6. The model output shows a significant effect of Grammaticality and Interference, suggesting that the grammatical condition is read significantly faster than both ungrammatical conditions, while the interference condition is read faster than the plain unlicensed condition. But, the model did not show any effect of CS scores, nor any interaction between CS scores and any other effects.

**Table 5:** **NPI licensing RTs: fixed effects from the maximal linear mixed effects model on the critical word and the spill-over word**.

	**CW**	**CW + 1**
	***p*-value**	***p*-value**
Grammaticality	<0.0001^***^	0.03^*^
CSscore	0.76	0.36
Interference	0.02^*^	0.33
Licensor	0.68	0.006^**^
Gram:CSscore	0.88	0.28
Inter:CSscore	0.92	0.42
Licensor:CSscore	0.12	0.9
Inter:Licensor	0.22	0.33
CS:Inter:Licensor	0.4	0.53

model = lmer[logRT ~ gram * CSscore + inter * CSscore + licensor * CSscore + licensor:inter + licensr:inter:CSscore + (1 + gram + licensor + inter | subj) + (1 + gram + licensor + inter | item), data = dataframe].

* p < 0.05;

** p < 0.01;

*** p < 0.001.

**Table 6:** **NPI licensing RTs: fixed effects from the linear mixed effect models, separated for two different licensors**.

	**Word CW**		**Word CW+1**	
	**Licensor No**	**Licensor Only**	**Licensor No**	**Licensor Only**
	***p*-value**	***p*-value**	***p*-value**	***p*-value**
Grammaticality	0.0005^***^	<0.0001^***^	0.01^*^	0.2
Interference	0.005^**^	0.15	0.5	0.1
CSscore	0.60	0.96	0.3	0.4
Gram:CSscore	0.35	0.53	0.4	0.1
Inter:CSscore	0.79	0.92	0.5	0.6

model = lmer(logRT ~ gram * CSscore + inter * CSscore + (1+ gram + inter|subj) + (1+ gram + inter + CSscore|item), data = dataframe).

* p < *0.05*;

** p < 0.01;

*** p < 0.001.

Although the interaction between CS scores and Interference shown in Table 6 isn't significant, to find out if there was any trend of an effect from CS scores, we carried out an exploratory correlation and split group analysis for the CW. The procedure was the same as with the analyses of acceptance rate data presented above. The first result is that there was no correlation between CS scores and interference [Pearson's *r* = −0.05, *t*_(86)_ = −0.49, *p* > 0.6]. For the split group analysis, we again separated participants into a high-CS (*n* = 36) and a low-CS group (*n* = 43) based on the median-split (CS = 3) of their CS scores. The mean RT for each group is plotted in Figures 5B,C. For the high-CS group, on the CW *ever*, neither the effect of Grammaticality nor Interference was significant (*p*s > 0.2)—there was no difference between any of the conditions (licensed, 433 ms; interference, 438 ms; unlicensed, 454 ms). For the low-CS group, however, both Grammaticality (*p* < 0.01) and Interference (*p* < 0.05) were significant. The licensed NPI (417 ms) was read faster than the unlicensed (480 ms) and the interference condition (433 ms); the interference condition was also faster than the unlicensed condition.

To summarize, on the CW, the averaged data showed the standard Grammaticality and Interference effect, but neither the mixed effects model nor the correlation analyses suggested any interaction between CS scores and the Interference effect. The split group analysis showed a small trend of modulation by CS scores: only the low-CS-scores group showed Interference, but it is difficult to draw any conclusions from this result since the high-CS-scores group did not show any difference between conditions, let alone an interference effect.

***On the word CW + 1.*** On the spillover word (Figure 5A), the grand average over all the participants showed a faster reading time on the licensed condition (407 ms) than on the interference and unlicensed conditions (both were 434 ms). There is a significant Grammaticality effect, but no effect of Interference, CS scores, or any interactions (see Table 6). The exploratory correlation analysis found no correlation between the size of the interference effect and the CS scores [Pearson's *r* = −0.07, *t*_(86)_ = −0.7, *p* > 0.4]. The exploratory split-group analysis in which we separated the high-CS and low-CS groups of participants, however, revealed different trends for the two groups (see Figures 5B,C). For the high-CS group, there was an effect of Grammaticality (*p* < 0.01), but no Interference (*p* > 0.6). The licensed condition (410 ms) was read faster than both the interference (450 ms) and the unlicensed conditions (446 ms), and there was no difference between the latter two. For the low-CS group, there was no effect of Grammaticality or Interference (Grammaticality, *p* > 0.2; Interference, *p* > 0.5; licensed, 402 ms; interference, 428 ms; unlicensed, 413 ms).

To summarize the results for the licensor *no*, grand average data showed a significant Grammaticality effect and an Interference effect on the CW. For the spillover word, there was only a Grammaticality effect. However, when we separated the high-CS group from the low-CS group, there was a trend of an effect of CS scores: for the high-CS group, there was no difference at the CW, but there was a grammaticality effect at the spill-over word, without an interference effect; for the low-CS group, there was both a grammaticality and an interference effect on the CW, yet no differences at the spill-over word. In other words, the low-CS group showed immediate sensitivity to ungrammaticality at the critical NPI word, but this sensitivity is also prone to an interference effect; the high-CS group, on the other hand, was slightly delayed in showing sensitivity to ungrammaticality, but, at the same time, was more resistant to the interference effect. Some caution is warranted, however, in interpreting the results from the split-group analysis, since based on the comprehensive model and the exploratory correlation analysis, the interaction between CS scores and Interference essentially presented a null result.

#### Licensor only

**Figure 6:**
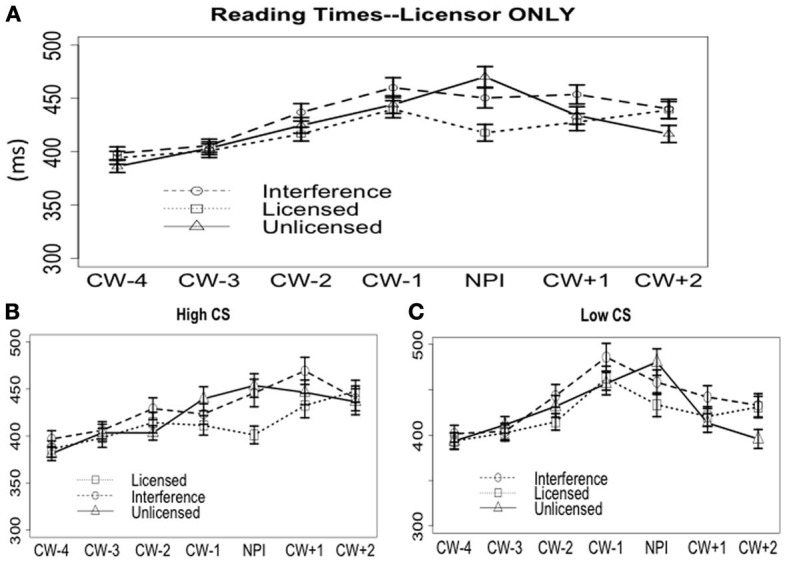
**Reading times when the NPI licensor was “*only.*” (A)** Word-by-word reading time cross all subjects. **(B)** Word-by-word reading time for the high CS group. **(C)** Word-by-word reading time for the low CS group.

***On the word CW.*** The grand average of the word-by-word RTs are shown in Figure 6A. On the CW *ever*, the licensed condition (420 ms) was read faster than the plain unlicensed condition (473 ms) and the interference condition (453 ms). The model output in Table 6 shows only a significant Grammaticality effect, but no Interference effect. This is significantly different from licensor *no*, and we will discuss it further in the general discussion.

The results from the exploratory correlation analysis found no correlation [Pearson's *r* = 0.03, *t*_(86)_ = 0.3, *p* > 0.7]. Results from the split group analysis are shown in Figures 6B,C. For the high-CS group, there was an effect Grammaticality (*p* < 0.001), but no effect of Interference (*p* > 0.4): the licensed condition (401 ms) was read significantly faster than both the interference condition (446 ms) and the unlicensed condition (454 ms), and there was no difference between the latter two. For the low-CS group, there was also an effect of Grammaticality (*p* < 0.01). There seems to be a numerical trend of interference, but the effect of Interference wasn't significant (*p* > 0.2) (licensed 434 ms; unlicensed 480 ms; interference 458 ms).

***On the word CW + 1.*** On the spillover word, the grand means (Figure 6) of the three conditions are: licensed 428 ms, interference 454 ms, and unlicensed 434 ms. The comprehensive mixed-effect model did not reveal any significant effects of Grammaticality or Interference (Table 6), and this was confirmed by the mixed effect models within each CS group (all *p*s > 0.1).

To summarize the self-paced reading time data from the licensor *only*, the grand average data showed a grammaticality effect without an interference effect. The same pattern largely holds for both high and low-CS groups, but the low-CS group showed a small trend of interference, as well.

#### Subject-verb number agreement

Within the subject-verb number agreement materials, two Helmert contrasts were defined in order to examine both the grammaticality effect: ***Grammaticality*** (10a vs. 10b and 10c in Table 1), and the interference effect: ***Interference*** (10b vs. 10c). Everything else about the mixed effects model structures was set up in the same way as for the analyses of NPI licensing.

#### Acceptability rating

The average acceptability rating (see Table 2) was 0.92 for the grammatical condition (10a), 0.12 on the plain ungrammatical condition (10c), and 0.28 on the interference ungrammatical condition (10b). The mixed effects logistic model showed a significant Grammaticality effect and a significant Interference effect. No other effects were significant. Crucially different from the NPI results (see Tables 3, 4), CS scores did not affect participants' judgment of subject-verb agreement errors. The model output for the fixed effects is shown below in Table 7.

**Table 7:** **Number-agreement acceptance rate: fixed effects from the mixed effect logistic model**.

	**Estimate**	**Std. Error**	***z* value**	***Pr*(>|*z*|)**
Grammaticality	1.99	0.2	9.4	<2e-16^***^
Interference	0.82	0.15	5.59	2.3e-08^***^
CSscore	0.09	0.07	1.25	0.2
Gram:CSscore	0.04	0.06	0.68	0.5
Inter:CSscore	−0.02	0.04	−0.61	0.5

model = lmer(acceptance ~ gram * CSscore + inter * CSscore + (1+ gram + inter|subj) + (1+ gram + inter + CSscore|item), data = dataframe, family = “binomial”).

*** p < 0.001.

**Figure 7:**
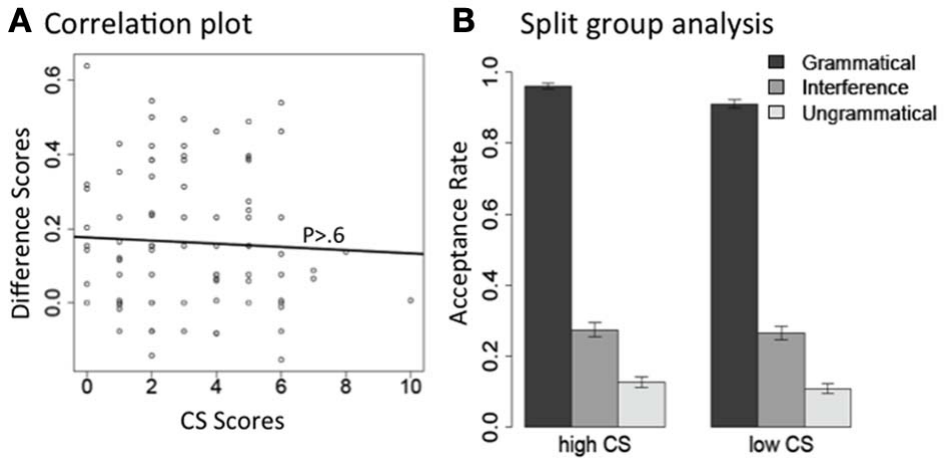
**The interaction between interference effect and CS scores in acceptance rate, for subject-verb agreement errors. (A)** Correlation between each individual subject's difference scores between their interference condition and the plain unlicensed condition (*Y*-axis: acceptance rate of Interference—acceptance rate of Ungrammatical) and their CS scores (*X*-axis: CS scores). **(B)** Acceptance rate for each condition plotted separately for the high and low CS groups.

To make a parallel comparison with the NPI stimuli, Figure 7A presents the correlation between the interference effect and CS scores, and Figure 7B presents the median-split analysis. The lack of correlation in Figure 7A (Pearson's *r* = 0.05, *p* > 0.6) confirms that CS scores did not affect the interference effect in the agreement items. And the mixed effect models within each group also found the same Grammaticality and Interference effects for both high and low-CS groups (all *p*s < 0.0001).

#### Self-paced reading time

**Figure 8:**
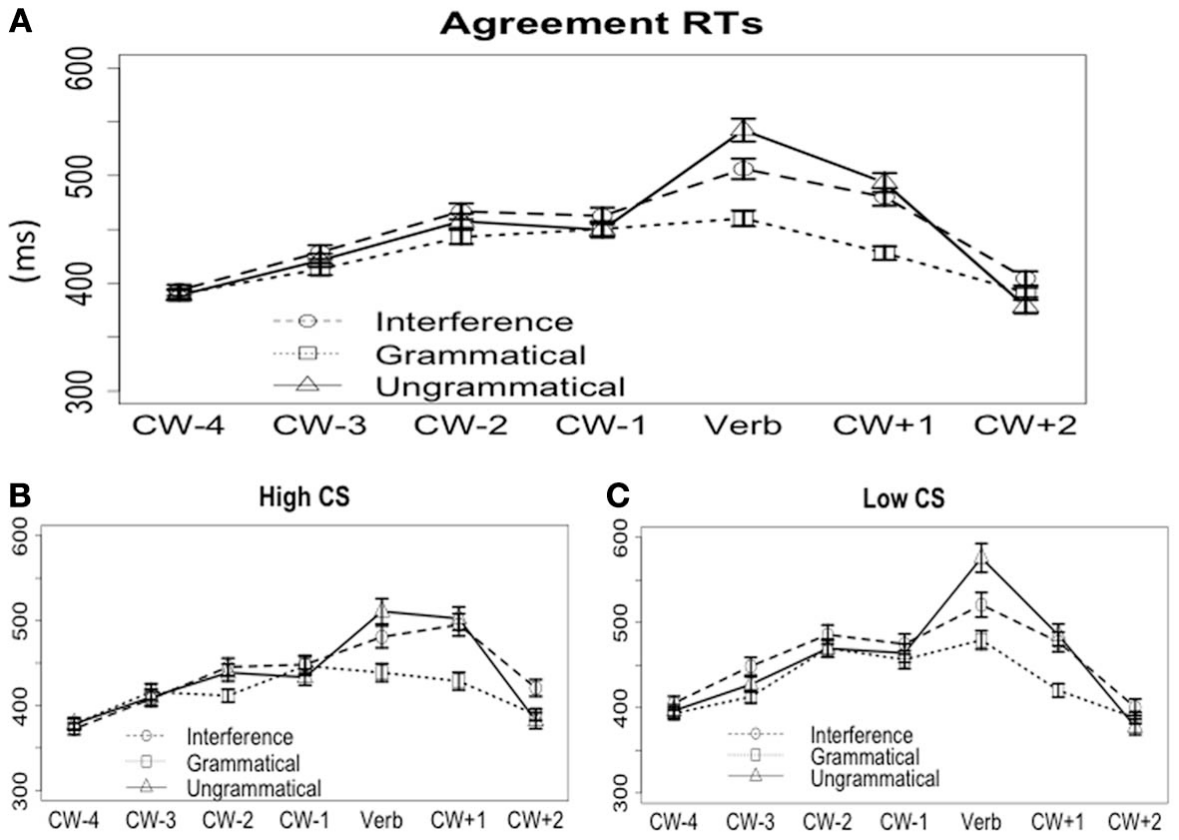
**Reading times for the agreement set of the stimuli. (A)** Word-by-word reading times across all subjects. **(B)** Word-by-word reading times for the high CS group. **(C)** Word-by-word reading times for the low CS group.

***On the word CW.*** Word-by-word reading times are plotted in Figure 8A. The average RTs on the critical verb (e.g., *fail* in example 10b) are 463 ms for the grammatical condition (10a), 547 ms for the plain ungrammatical condition (10c), and 509 ms for the interference condition (10b). The mixed effects model showed a significant effect of Grammaticality and Interference (see Table 8) on the CW, such that the grammatical condition was read faster than the other two conditions (*a* vs. *b, c, p* < 0.001), and the interference condition was read faster than the plain ungrammatical condition (*b* vs. *c, p* < 0.01). There were no interactions between Interference and CS scores.

**Table 8:** **Number agreement RTs: fixed effects from the linear mixed effect model**.

	**Word CW**	**Word CW + 1**
	***p*-value**	***p*-value**
Grammaticality	<0.0001^***^	<0.0001^***^
Interference	0.01^*^	0.5
CSscore	0.5	0.6
Gram:CSscore	0.6	0.5
Inter:CSscore	0.6	0.6

model = lmer(logRT ~ gram * CSscore + inter * CSscore + (1+ gram + inter|subj) + (1+ gram + inter + CSscore| item), data = dataframe).

* p < 0.05;

*** p < 0.001.

The split-group analysis, as shown in Figures 8B,C, revealed qualitatively similar patterns for high-CS and low-CS groups. For the high-CS group, there was an effect of Grammaticality (*p* < 0.001): the grammatical condition (439 ms) was read significantly faster than both the ungrammatical condition (511 ms) and the interference condition (481 ms); and an effect of Interference as well (although slightly weaker, *p* < 0.07). For the low CS group, the grammatical condition (480 ms) was read significantly faster than both the ungrammatical condition (576 ms) and the interference condition (521 ms); and the difference between the latter two was also significant (Grammaticality, *p* < 0.01; Interference, *p* < 0.01).

#### On the word CW + 1

At the spillover word, grand averages of the three conditions were: grammatical 428 ms, interference 480 ms, and ungrammatical 493 ms. The mixed effects model showed a significant effect of Grammaticality, but no effect of Interference (see Table 8). The split-group analysis (see Figure 8) revealed very similar results for both participant groups. For the high-CS group, the grammatical condition (429 ms) was read faster than the ungrammatical condition (502 ms) and the interference condition (500 ms), and there was no difference between the latter two (Grammaticality, *p* < 0.0001; Interference, *p* > 0.8). The low-CS group showed the same pattern: the grammatical condition (420 ms) was read faster than the ungrammatical (485 ms) and interference (477 ms) conditions (effect of Grammaticality, *p* < 0.001), with no significant difference between the latter two (effect of Interference, *p* > 0.8).

To summarize, for the agreement stimuli, we observed the grammaticality effect and the interference effect in both acceptability ratings and the self-paced reading time on the critical word. On the spillover word, self-paced RTs only showed a grammaticality effect, but no interference. In all these measures, the high and low-CS participants performed in very similar ways.

## General discussion

The current study revealed three main findings. First, only NPI interference, but not agreement interference, is affected by individual subject's pragmatic-communicative skills. Second, the modulation of pragmatic-communicative skills mostly has its effect on offline acceptability rating, but not online reading time, although there seems to be a trend of effect in online RTs as well. And third, different NPI licensors, in particular, *no* and *only*, presented distinct interference profiles: while both showed offline interference in acceptability, NPIs under *only* did not show online interference. We turn below to the discussion of these observations.

### Interference in acceptance rate and the effect of autistic traits

A critical finding of the current study is that for the NPI materials, but not for the agreement interference stimuli, participants' acceptance rate was affected by their autistic-associated traits; in particular, their communication skills, as measured by the CS of the AQ questionnaire. Participants with higher CS scores, i.e., those that are relatively worse in their general pragmatic communicative skills, were less prone to NPI interference, as demonstrated by their more accurate acceptability judgments. On the other hand, participants with better communicative skills (lower CS scores) more often accepted the interference conditions. In contrast to the case with NPI licensing, participants' autistic traits did not seem to affect their acceptance of subject-verb agreement sentences, suggesting that subject-verb interference and NPI interference, although on the surface they look very similar, may arise from different sources.

We argue that the different interference profiles stem from the fact that NPI licensing and subject-verb agreement are different types of linguistic dependencies. It is uncontroversial that subject-verb number agreement involves a syntactic matching process that checks the number features on the subject and its corresponding verb. In incremental parsing, the subject of a sentence is likely to have been removed from focal attention when the verb is encountered (McElree, 2001); therefore, the real-time construction of a subject-verb agreement relationship depends on the successful retrieval of the subject's features. Memory-retrieval-based interference arises when the target of retrieval shares certain features with other items that have recently been processed (Lewis and Vasishth, 2005; Lewis et al., 2006). Under this account of subject-verb agreement and the corresponding interference effect, interference errors stem from misapplication of the mechanism by which number agreement is computed.

We likewise argue that NPI interference is closely tied to one of the mechanisms by which NPIs are regularly licensed. As discussed in the introduction, in addition to a logical-semantic mechanism, there is also a pragmatic component to NPI licensing in English. Particularly relevant to our purposes here, negative inferences are employed regularly as part of a pragmatic licensing mechanism for NPIs. During the comprehension process, the parser may over-apply the pragmatic licensing strategy, and use even unwarranted negative inferences to license NPIs, resulting in interference.

Under this account, interference in syntactic agreement and interference in NPI licensing are driven, at least partially, by different underlying sources. This shouldn't be totally unexpected, since these two linguistic phenomena involve different representations and computations in the first place: the agreement process is purely syntactic, whereas NPI licensing is at the interface of different systems, including syntax, semantics, and pragmatics. It is not surprising that the specific linguistic properties of each construction lead to substantial differences in how they are processed in comprehension.

The current results also add to the growing literature that autistic traits are present among the neurotypical population and they affect language processing in non-trivial ways. Our results, in line with previous findings, suggest that the two sub-scales from the AQ—Social Skill and Communication—may have particular influence on pragmatic language processing. Since case studies in this regard are still relatively sparse, more future research is needed to further establish this association. There are many different kinds of pragmatic phenomena in language processing, and it is an open question whether they are in general affected by individual differences along the dimension of autistic traits. If it turns out that autistic traits only selectively target a subset of these phenomena, it would be very informative for the construction of a constrained pragmatic theory of language processing.

### Online interference and the (lack of) effect of autistic traits

Although there is a strong effect of autistic traits on the offline acceptability rating, their effect on online reading time is much weaker. The split group analysis seems to show a trend where there is more interference for the low-CS group than the high-CS group, but the mixed effects models revealed no interaction between CS scores and interference effect. The lack of an interaction in the comprehensive model could be due to insufficient power in the data, in which case we may still consider the online interference effect as being qualitatively similar to the offline effect. This is a potential explanation, but also one that is difficult to validate given the null result. While keeping this possibility in mind, we will entertain the alternative possibility that there is genuinely no effect of CS scores on online interference and discuss the implications of that possibility. Another interesting observation about the online interference effect is that, for NPI licensing, we only observed interference for the licensor *no*, but not the licensor *only*. The difference between these two licensors is important for our explanation of the online interference effect, but we will focus only on *no* for the moment, and come back to *only* in the next section.

The lack of modulation by participants' communicative-pragmatic skills on the online interference effect suggests that NPI licensing may actually involve a syntactic matching process, like subject-verb agreement. This was the original hypothesis in Vasishth et al. (2008), which postulated a search process for a syntactic [+Neg] feature when an NPI word such as *ever* is encountered. We questioned this hypothesis earlier because it does not fully represent how NPIs are licensed—it overlooks the fact that NPI licensing is not *just* a syntactic process, but involves semantic and pragmatic mechanisms. However, the fact that NPI licensing is an interface phenomenon that involves multiple levels of representations and processes does not exclude the possibility that syntactic matching exists within one sub-component of the licensing process. The syntactic [+Neg] feature is a particularly suitable candidate to serve as the relevant matching feature, since, cross-linguistically, negation is the most robust NPI licensor. This line of reasoning would make NPI licensing similar to subject-verb agreement in some respects. If the regular memory retrieval mechanisms apply in both cases, one would expect similar online interference with no modulation from individual pragmatic skills.

We also want to point out that, by recognizing such a syntactic licensing process, at least for licensors such as *no* (see the contrast with *only* below), we acknowledge a syntactic process for NPI licensing that has not been fully recognized or emphasized in previous research for weak NPIs like *ever*, which can be licensed under a broad range of licensors. Polarity items that are only licensed by negation—called “strict” NPIs (Giannakidou, 1998, 2011; Zwarts, 1998)—are cross-linguistically common, for example, so-called “n-words” in Romance languages. Purely syntactic mechanisms like agreement have been proposed to account for the distribution of n-words (Haegeman and Zanuttini, 1991; Zanuttini, 1991; Zeijlstra, 2004, inter alia). But, traditionally, the general account of NPI licensing, especially for weak NPIs like *ever*, has been deliberately divorced from an agreement-based explanation. We agree with the traditional wisdom, but based on the current data, we also suggest that a syntactic feature matching process may exist in parallel with other licensing mechanisms, even for weak NPIs, at least for a subset of licensors—those that contain a syntactic [+Neg] feature.

Licensing as an integrated syntax-semantics process is to be expected if (a) we take seriously the idea that NPI licensing is a grammatical phenomenon driven by the logical properties of lexical expressions, and (b) there is a strict isomorphism between the syntax and the semantics. Under these two theses, the logical property of negation is mapped onto a morphosyntactic feature [+negative] (for an early discussion of such a model see Giannakidou, 1998). NPI licensing will then always involve at least this component of integrated syntax-semantics matching, and online processes access that. But importantly, even if we recognize a syntactic feature-matching component in NPI licensing, the overall process is still crucially different from subject-verb agreement in many ways. In particular, NPI licensing involves semantic *and* syntactic, as well as pragmatic mechanisms, as we discussed earlier. But for the agreement sentences, whether they are acceptable or not is determined only by whether or not the syntactic matching process on the relevant number feature is successful—there is no obvious connection between the processing of syntactic agreement and the final interpretation of a sentence. For instance, Lau et al. (2008) showed that when people were lured by interfering agreement number features, as in “*The phone by the toilets were …*,” they nevertheless did not make mistakes in assigning the correct thematic role to the subject. NPI licensing, on the other hand, is a very different phenomenon. The presence of an NPI makes important contributions to the final propositional content. The acceptability of an NPI is not determined by the syntactic matching process alone, but is instead crucially regulated by semantic and pragmatic integration conditions. The effect of pragmatic inferences could be particularly strong in offline tasks, since participants are given enough time to reflect on what the target stimulus actually means, or could have meant.

The strong offline effect of individual subject's pragmatic skills leads to the question why such effects did not surface in online interference. One possibility is that the influence of pragmatic factors in online measures could have been masked by the strong presence of the memory-retrieval based effect, and hence was undetectable. This is not the most likely hypothesis, since for the licensor *only*, which we argue did not participate in a memory-retrieval based interference effect, we still did not observe a pragmatically driven interference in online measures. The other possibility is that since these interference sentences are ultimately ungrammatical, the pragmatic inference may be a “last resort” strategy” in these situations, and hence have a delayed effect. We discuss these issues in more details in the next section, together with data from the licensor *only*.

Our current discussion about the source of online interference for *no* departs somewhat from our earlier work in Xiang et al. (2009), in which we conjectured that even the online interference effect for *no* (and *few*) was driven by pragmatic processes. In the current discussion we draw a distinction between online and offline interference effects, and argue that since a syntactic matching process is possible between *no* and an NPI, (some) online interference may thereby arise through a memory retrieval process, as argued in Vasishth et al. (2008) (modulo the possible additional contribution of a pragmatic process, as shown by the trend in the split group analysis). However, one important feature of the original analysis in Vasishth et al. (2008) is that memory search targets positions that are ruled out on syntactic grounds, and that NPI interference under *no* is a demonstration of syntactic interference, as accessing a licensor in a non-commanding position purportedly violates syntactic constraints. We do not think that the current results necessarily commit us to this position. We contend that questions about the search mechanism (i.e., whether or not the search process is blind/insensitive to syntactic constraints) and whether or not NPI licensing shows similarity-based feature interference may be two orthogonal issues. Although NPIs are generally c-commanded by their licensors, it is not obvious that a c-command requirement should be stated explicitly as part of the syntactic requirements on NPI licensing. It could simply be an epiphenomenon, within an isomorphic syntax-semantics level, of the semantic requirement that an NPI needs to stay in the semantic scope of its licensor. The computation of the semantic scope may track configurational relations like c-command, but this does not necessarily mean that the parser actually makes reference to the c-command condition in online processing. In other words, the memory retrieval process may target a [+negative] element, instead of a [+negative, +c-command] element, while there is simultaneously a semantic condition that checks whether or not the NPI falls within the semantic scope of the retrieved target. Of course this leaves open a number of non-trivial questions as to how semantic scope is tracked in online processing. One possibility is that we encode [+scope] in some way as a lexical feature on the retrieval target, and interference would arise largely in the same fashion as the proposal in Vasishth et al. (2008), but with the syntactic feature [+c-command] replaced by the semantic feature [+scope]. This approach calls for a detailed implementation as to how scope relations could be encoded as lexical features, when they obviously are not features stored in the lexicon. The other possibility is that scope relation can only fall out while propositional content is being incrementally composed, rather than being encoded on lexical items. If this is true, we need an explicit algorithm that can both derive correct scope relations at the proposition level, and also allow incorrect scope relations to be derived, in order to account for the interference effect. We do not have answers to these questions. But we think it would be too hasty to reach a conclusion about the exact search mechanism involved in NPI interference without fully exploring all of these logical possibilities.

### Different types of NPI licensors

The perspective that multiple mechanisms are acting in parallel to license NPIs also helps explain the difference observed between the licensors *no* and *only*.

As discussed above, there is consensus that *only* does not license NPIs through a lexically encoded (syntactic) [+negative] feature, though the exact licensing mechanism for *only* as an NPI licensor is still under debate. The difference between *only* and other negative licensors such as *no* (or *few*) can be demonstrated by the syntactic diagnostics provided in Klima (1964), as was illustrated earlier.

In the current results, we saw that on grammatical conditions, NPIs licensed under *only* were accepted less often than those licensed under *no* (Figure 1). This could be due to a number of factors. For instance, *no* is a more frequent licensor than *only* in naturally occurring utterances (Xiang et al., 2009). This may have influenced the acceptability ratings of the two licensors. Alternatively, under the licensor *no*, an NPI can be licensed both syntactically and semantically. Syntactically, a feature-matching process may search and identify a target with a [+Neg] feature; semantically, a negative meaning may also be calculated. Syntactic and semantic processes converge on the final representation in which an NPI is licensed. With the licensor *only*, however, the syntactic feature-matching process fails, since *only* does not contain a morphosyntactic feature [+Neg]. Then, only the semantic route (via the exceptive entailment “nobody other than”) would be available. The failure of isomorphism between syntax and semantics, in contrast with *no,* may have reduced the acceptability of NPIs under *only*. In a recent study (Xiang et al., 2013), acceptability ratings were collected for a larger set of licensors, including *no, few, only*, and emotive factives such as *amazed, surprised*, etc. It was found that the two syntactically negative licensors *no* and *few* are judged more acceptable than *only* and emotive factives, which are both non-negative. This is completely in line with the results reported here. Furthermore, since *few* is also much less frequent than *no* as an NPI licensor (Xiang et al., 2009), this result also suggests that lexical frequency *per se* does not completely determine the degree to which a licensor is accepted.

It is worth noting that the current study also revealed some difference between *no* and *only*: there was no obvious online interference effect for *only* (modulo the possibility that the low-CS group may have shown a trend of online interference). In a previous self-paced reading study, Xiang et al. (2006) also showed a lack of interference effect in online RTs for *only*. As mentioned earlier, the interference effect of *only*, or the lack of one, has not been widely tested. Although the current results showed a difference between *no* and *only* when we analyzed these two licensors separately (Table 6), there wasn't a Licensor by Interference interaction in the overall model in Table 5. This could be due to insufficient power in the data. We are fully aware that the lack of an interaction may undermine our proposed account of *only* here. More studies are needed to verify whether or not *only* is indeed resistant to online interference. But if it is, such a result is completely in line with the distinction we draw here between *no* and *only*: the former, but not the latter, is targeted by a syntactic feature-matching process. Therefore, similarity-based interference, which crucially relies on specific lexical features, will not arise for *only* online. The immediate question for this explanation is why pragmatically-driven interference does not appear online.

One possibility is that the pragmatic inferences that drive the interference effect cannot be generated in time to trigger online interference. Instead, they are delayed until a later stage, and therefore only offline tasks can detect them. This is not the most likely hypothesis, however, given the large literature that has suggested that pragmatic inferences can be incrementally generated online (Altmann and Steedman, 1988; Sedivy et al., 1999; van Berkum, 2009; Nieuwland and Kuperberg, 2008). We propose that the reason pragmatically-driven interferences were predominately observed offline in the present study is not that such inferences failed to become available in time, but rather that the available inferences were not immediately adopted by the comprehension system to license NPIs.

First of all, the pragmatic licensing mechanism could in general be a more costly strategy than regular syntactic and semantic mechanisms. In a recent ERP study, Xiang et al. (2012, 2013) showed that, even for grammatically acceptable sentences, there is difference between NPIs that are licensed under pragmatically-derived negation (e.g., the negative implicature from emotive factive predicates) and those that are licensed under regular semantic negation (such as *no*)—only the semantic negation, but not the pragmatic negation, had a small P600 compared to the ungrammatical control condition. Second, as we mentioned above, while we recognize that pragmatically-derived negative implicatures can license NPIs, we also recognize that not all implicatures can do so. The specific conditions characterizing the “usable” implicatures are yet to be isolated, but we have conjectured that the kind of pragmatic implicatures that trigger interference effects are normally insufficient to actually license NPIs. It is likely that the comprehension system does not resort to such implicatures unless it is pushed into a corner, as in the presence of an ungrammatical sentence. If pragmatically-driven interference were the result of a last-resort strategy, it wouldn't necessarily surface in online processing.

### A multi-dimensional system of NPI licensing

NPI licensing reveals a case in which syntactic, semantic, and pragmatic processes act in parallel during parsing, which makes NPI licensing qualitatively different from purely syntactic dependencies, such as subject-verb agreement. The processing profile of NPI licensing is therefore much more complicated. Some licensors, such as *no*, which can participate in a syntactic licensing relation with an NPI, may be targeted by the same memory retrieval mechanisms that target other syntactic dependencies; but, for other licensors that do not bear the relevant syntactic features, memory retrieval of a lexical feature does not apply. In addition, since pragmatic licensing is a regular mechanism for NPI licensing, at least for English weak NPIs, the comprehension system may stretch it to cases in which pragmatic licensing normally does not apply, leading to pragmatically-driven interference.

To account for the full complexity of NPI licensing and the interference effect associated with it, a number of open issues need to be addressed in future work. First of all, if, as we argued above, interference associated with feature similarity only arises for licensors that contain a lexical [+negative] feature, we predict that online interference should be observed for some NPI licensors, but not others. Expressions that can license NPIs and, at the same time, are categorized as real negative expressions (i.e., under Klima, 1964) include *no, none, not, never, few, hardly, scarcely, seldom*, etc.; on the other hand, licensors that are not negative in the regular sense include the examples we mentioned earlier, such as *only, every*, comparatives, conditionals, emotive factives, questions, etc. Neither of these two groups has been tested exhaustively.

Second, we have argued that NPI interference is partially driven by pragmatic inferences, especially in the case of offline interference. We have made suggestions both about how such inferences arise and why they seem to be more prominent in offline measures. Our account of pragmatic interferences is closely associated with a particular construction that has been heavily tested by other researchers, as well as in our current work—that is, relative clauses. We made use of the well-known fact that modifiers, with relative clauses as a prime example, invite contrastive inferences. This gives rise to the following prediction: complement clauses (such as “*The fact that no student passed the exam …*”), which are minimally different from relative clauses but do not serve a modifier function, should not show interference effects, or at least not the kind of interference effect we have shown that can be modulated by individual subjects' pragmatic skills. Some results from Parker and Phillips (2011) provide preliminary support for this prediction. These authors showed a reduced interference effect for complement clauses, compared to relative clause structures, under the licensor *no*. Furthermore, with a licensor like *only* (as in “*The fact the only the best students passed the exam …*”), we predict that the interference effect on such clauses should be reduced to minimum, since the pragmatic source of interference has been entirely eliminated by the complement clause, and, in the meantime, “*only*” does not trigger syntactically associated interference either.

Finally, most of the current work on NPI interference has focused on languages that allow weak NPIs. These have a broad distribution and can be licensed under a variety of licensors. We conjectured that, for such NPIs, an independently available pragmatic licensing mechanism is over-applied in some situations, resulting in interference. Cross-linguistically, however, many languages have NPIs that are much more restricted in their distribution. It is possible that for some of these stricter NPIs, pragmatic-licensing mechanisms are never available in the grammar. This territory—interference with such NPIs—is still largely uncharted (for a recent examination of this sort, see Yanilmaz and Drury, 2013)^9^; and if they do show interference, we predict it to be syntactically-driven interference, and not to be subject to individual differences in pragmatic skills.

## Conclusion

In this study, we compared the interference effects in syntactic agreement and NPI licensing, especially with respect to their modulation by individual subjects' pragmatic skills. We showed that the interference profile for NPI licensing is more complicated than that for syntactic agreement, due to their representational differences. In particular, NPI interference is affected by (a) the type of NPI licensors involved, (b) the particular experimental tasks, and (c) individual subjects' pragmatic-communicative skills. All together, our results show that NPI licensing, different from the pure syntactic processes involved in agreement, evokes multiple different processes corresponding to different levels, or dimensions, of linguistic representations.

### Conflict of interest statement

The authors declare that the research was conducted in the absence of any commercial or financial relationships that could be construed as a potential conflict of interest.

## Footnotes

^1^Cue similarity does not always lead to facilitation effect. For instance, Van Dyke and Lewis (2003) and Van Dyke and McElree (2006) have shown that cue overload leads to increased processing difficulty. In this paper, we only focus on the facilitatory interference effect.

^2^Some NPIs, such as any, seem to obtain so-called *free choice* readings in modal environments and with imperatives, such as *You may talk to any student*, and *Pick any card*!. We won't discuss the free choice use in this paper. The particular NPI *ever* studied in our experiment, does not have a free choice use and it is typically blocked in modal contexts: ^*^*You may ever go to Paris.*

^3^A scalar component has also been posited for some NPIs (Kadmon and Landman, 1993; Krifka, 1995; Lahiri, 1998; Chierchia, 2006), but scalarity doesn't characterize *all* NPIs as a class. There are many non-scalar NPIs (see Giannakidou, 1998, 2011, for a recent overview (Lin, 1996; Giannakidou and Yoon, 2012). And scalar NPIs such as *any* and *ever* do not have only scalar uses (Duffley and Larivée, 2010), and are not morphologically marked as scalar either, i.e., they do not contain scalar markers such as *even* and the like. Whether scalarity plays a role in the interference effect is an open question for future research.

^4^There is ongoing discussion on these expressions. For *only* and *emotive factives*, von Fintel (1999) and Gajewski (2005) analyzed them as Strawson DE, rather than regular DE expressions. Limited by space, we won't discuss further about this possibility, but see theoretical and experimental evidence for problems of this approach in Linebarger (1987), Giannakidou (2006) and Xiang et al. (2012, 2013). We also won't go into further discussion about *long after*, since it is not the focus of this paper, but see Condoravdi (2010) that proposed a DE analysis, and also Krifka (2010) for a discussion.

^5^Although we adopt the term “implicature” here, it should be clear from our discussion that we are aware this concept is still very vague. Not all negative implicatures can license NPIs. The exact grammatical constraints and mechanisms that rule in some “implicatures” but rule out others remain as an open question.

^6^See Horn (2002) for the idea and the negative component in “almost” is “assertorically inert,” in contrast to “barely,” and hence does not license NPIs.

^7^Among the other subscales, social skill showed a very similar effect as CS. Other subscales did not show any effect. We report the interactions between these sub-scales and other fixed effects in the appendix.

^8^The model in Table 3 did not include all the interaction terms between the three user-defined contrasts. Note that the interaction between Grammaticality and Interference is irrelevant, since there is no interference to start with on the grammatical conditions (i.e., interference conditions are themselves all ungrammatical). For the interaction between Licensor and Grammaticality/Interference, since our experimental design is not a full 2 × 3 factorial design (i.e., there is only one plain unlicensed condition in Table 1), not all possible interaction combinations are possible. Therefore, we only included Licensor:Interference in the regression formula, which is essentially the same as the Licensor:Grammaticality interaction.

^9^The recent ERP findings in Yanilmaz and Drury (2013) tested Turkish NPIs that have a very limited distribution. Interference was found on these NPIs. Since the constructions tested there were very different from the ones tested here (sentential complement of a matrix verb was tested), we won't go into further details. But the additional novel factor in Yanilmaz and Drury (2013) is that NPIs in Turkish come before their licensors in linear order, which may result in a forward expectation of a licensor (e.g., similar to a regular filler-gap dependency), rather than just backward search, as in the case of English NPIs.

## Appendix

The interactions between the other four sub-scales from AQ and each of the fixed effect predictors, for NPI licensing and number agreement separately. The mixed effect models are constructed in similar ways as the models in Tables 3, 5, 7, 8. As shown below, the only significant effect observed is the interaction between the Social Skill sub-scale and the offline NPI interference effect (i.e., acceptance rate). This is similar to the effect of the Communication sub-scale. No other interaction was observed.

**Table 9:**

	**NPI licensing**			**Number agreement**	
**Acceptance rate (*p*-value)**	**Interaction with Interference**	**Interaction with Licensor**	**Interaction with Grammaticality**	**Interaction with Interference**	**Interaction with Grammaticality**
Social skill	0.006^**^	0.14	0.89	0.16	0.96
Attention to details	0.44	0.17	0.92	0.16	0.73
Attention switching	0.98	0.9	0.63	0.13	0.56
Imagination	0.15	0.44	0.94	0.61	0.096
**RT at the critical word (*t*-value)**	**Interaction with Intrusion**	**Interaction with Licensor**	**Interaction with Grammaticality**	**Interaction with Intrusion**	**Interaction with Grammaticality**
Social skill	−1.28	0.65	0.97	0.43	0.56
Attention to details	0.58	0.89	−0.74	0.65	−1.02
Attention switching	−1.46	0.98	0.54	0.86	0.54
Imagination	0.13	0.57	0.07	0.54	0.17

** p < 0.01.
